# SYT7 regulates the progression of chronic lymphocytic leukemia through interacting and regulating KNTC1

**DOI:** 10.1186/s40364-023-00506-4

**Published:** 2023-06-06

**Authors:** Wenjie Zhang, Jinlan Long, Peixia Tang, Kaili Chen, Guangyao Guo, Zezhong Yu, Jie Lin, Liping Liu, Rong Zhan, Zhenshu Xu

**Affiliations:** Fujian Provincial Key Laboratory on Hematology, Fujian Institute of Hematology, Fujian Medical University Union Hospital, 29 Xinquan Rd, Fuzhou, 350001 China

**Keywords:** Chronic lymphocytic leukemia, SYT7, KNTC1, Disease progression

## Abstract

**Background:**

Chronic lymphocytic leukemia (CLL) is one of the most frequent occurring types of leukemia. It typically occurs in elderly patients and has a highly variable clinical course. At present, the molecular mechanism driving the pathogenesis and progression of CLL is not fully understood. The protein Synaptotagmin 7 (SYT7) encoded by the SYT7 gene has been found to be closely related to the development of various solid tumors, but its role in CLL is unclear. In this study, we investigated the function and molecular mechanism of SYT7 in CLL.

**Methods:**

The expression level of SYT7 in CLL was determined by immunohistochemical staining and qPCR. The role of SYT7 in promoting CLL development was verified by in vivo and in vitro experiments. The molecular mechanism of SYT7 in CLL was elucidated by methods such as GeneChip analysis and Co-immunoprecipitation assay.

**Results:**

Malignant behaviors such as proliferation, migration, and anti-apoptosis of CLL cells were significantly inhibited after SYT7 gene knockdown. In contrast, SYT7 overexpression promoted CLL development in vitro. Consistently, the knockdown of SYT7 also inhibited xenograft tumor growth of CLL cells. Mechanistically, SYT7 promoted CLL development by inhibiting SYVN1-mediated KNTC1 ubiquitination. The KNTC1 knockdown also attenuated the effects of SYT7 overexpression on development of CLL.

**Conclusions:**

SYT7 regulates the progression of CLL through SYVN1-mediated KNTC1 ubiquitination, which has potential value for molecular targeted therapy of CLL.

**Supplementary Information:**

The online version contains supplementary material available at 10.1186/s40364-023-00506-4.

## Background

Chronic lymphocytic leukemia (CLL) is the most frequent lymphoproliferative disease in the western world [1]. It is characterized by the clonal expansion of CD5+/CD19+ malignant B cells that accumulate in the blood, bone marrow, lymph nodes, or other lymphoid tissues and shows a highly heterogenous pathophysiology with chromosomal aberrations, recurrent mutations, and microenvironmental involvement [2, 3]. Once treatment is indicated, many therapeutic options are available such as small molecule inhibitors, including Bruton’s tyrosine kinase (BTK) and B-cell lymphoma-2 (BCL-2) inhibitors, as well as standard chemoimmunotherapy [4]. Despite advances in management in recent years, treatment options for patients with CLL remain limited and are poorly tolerated. As a result of the highly heterogeneous clinical course of CLL there is a subset of treated patients who experience disease recurrence or progression in an extremely short time [5]. Many genetic alterations have been found to be closely related to the progression of CLL [6, 7], and there are emerging targeted agents leading the treatment of CLL into the era of precision medicine [8, 9]. But there are still many unknown reasons for the progress of CLL. The identification of novel targets is therefore still crucial for the prediction and treatment of CLL in the future [10].

The Synaptotagmins are a family of proteins identified as the most common multifunctional calcium sensors [11]. The Synaptotagmin 7 (SYT7) gene is located on human chromosome 11q12.2 and encodes a predicted single-pass 46-kDa transmembrane protein [12, 13]. SYT7 has been demonstrated to mediate diverse aspects of synaptic transmission including asynchronous neurotransmitter release, synaptic facilitation, and vesicle recruitment [14, 15]. SYT7 has also been implicated in many processes in non-neuronal cells such as mediating the secretion of the stress hormone, insulin, and other factors [16]. Previous studies have shown that SYT7 can promote the progression and liver metastasis formation of gastric cancer [17]. SYT7 has also been reported to promote the proliferation, invasion, metastasis, and the inhibition of cell apoptosis of non-small cell lung cancer cells [18]. Dong et al. clarified that SYT7 can promote thyroid cancer development by blocking BRCA1 mediated HMBG3 ubiquitination [19]. However, a role for SYT7 in CLL has not been reported.

In this study, we demonstrate that SYT7 plays an important role in driving the progression of CLL. In addition, we further explored the molecular mechanism, which may provide novel targets for precision treatment of CLL.

## Materials and methods

### Cell lines and Culture

Short Tandem Repeat (STR) qualified CLL cell lines MEC-2 and M01043 were purchased from the American Type Culture Collection (ATCC). All cells were maintained in RPMI-1640 medium (Gibco) containing 10% FBS at 37 °C with 5% CO_2_.

### Tissue microarray and Immunohistochemistry (IHC) analysis

Tissue microarrays for human non-Hodgkin’s lymphoma and normal lymph node were purchased from Xi’an Alena Biotechnology Co., Ltd. The CLL patients were completely informed before collecting the tissue samples and related clinical data. The experimental design in our study was approved by Ethics Committee of Fujian Medical University Union Hospital.

For IHC the tissue microarray samples were incubated at 60 °C for 30 min, then dewaxed in xylene and hydrated in graded alcohols. After blocking in 3% H2O2 for 5 min, the array chips were incubated with primary antibody (Table S1) at 4 °C overnight. After washing, the secondary antibody HRP Goat Anti-Rabbit IgG (Table S1) was added and incubated for 2 h at room temperature. For staining, the tissue specimens were immersed in DAB reagent, and counter-stained with hematoxylin. Images were exanimated and captured using a photomicroscope and analyzed with CaseViewer and ImageScope software. Specimens were classified into negative, positive, ++ positive, or +++ positive, based on the sum of the staining intensity (varied from weak to strong) scores and staining extent scores (Table S2). The median of the calculated total score was used as a cut-off value to dichotomize variables.

### Plasmid construction and lentivirus infection

The plasmid for overexpressing SYT7, SYVN1, and the negative control plasmid were designed by Shanghai Yibeirui Biomedical Science and Technology Co., Ltd. Three shRNA plasmids for SYT7 and KNTC1 and related negative controls were designed as well, and the sequences are shown in Table S3.

For cell transfection 2 × 10^5^ cells were cultured in a 6-well plate in 1640 medium with 10% FBS and lentiviral vectors (1 × 10^8^ TU/mL) were added and mixed with Enhanced Infection Solution (ENi.S) and Polybrene. Cells were cultured for 72 h until cell confluence reached 80%, then cells were harvested for subsequent experiments.

### qRT-PCR analysis

Total RNA from shRNA expressing lentivirus infected cells MEC-2 and M01043 were extracted using TRIzol reagent (Sigma). After quantification by a Nanodrop 2000/2000 C spectrophotometer (Thermo Fisher Scientific), 500 ng of RNA was used for cDNA synthesis. RT-PCR was performed with a Biosystems 7500 Sequence Detection system using a SYBR Green Mastermixs Kit (Vazyme). Relative gene expression was analyzed by the 2^−ΔΔCt^ method. Reactions were performed in triplicate and the corresponding primers used in PCR are detailed in Table S4.

### Western blotting (WB) assay and Co-immunoprecipitation (Co-IP) assay

Lysates from lentivirus infected MEC-2 and M01043 cells were prepared in ice-cold RIPA buffer (Millipore) and the concentration was determined using the BCA Protein Assay Kit (HyClone-Pierce). WB was performed using 20 µg proteins per lane. After separated by 10% SDS-PAGE (Invitrogen) and transfer onto PVDF membranes, the membranes were incubated with specific primary antibodies and appropriate secondary antibodies, antibody details are shown in Table S1. Protein bands were visualized by enhanced chemiluminescence (Amersham).

For co-immunoprecipitation assays, 1.0-1.2 mg total protein in lysis buffer was incubated with anti-KNTC1 or anti-SYT7 antibodies at 4 °C overnight. 20 µL magnetic beads were added and incubated for 2 h at 4 °C. After washing and centrifugation, the precipitates in 5 × loading buffer were boiled for 10 min and loaded onto 10% SDS-PAGE gels. The membrane was blocked with 5% skimmed milk, then incubated with primary antibodies and corresponding secondary antibody and the blots were visualized.

### Flow cytometry (FCM) for detecting cell apoptosis and cell cycle

FCM was used to visualize stages of the cell cycle and the extent of apoptosis of the lentivirus infected MEC-2 and M01043 cells. Apoptosis was determined after cell confluence reached 80%. The cells were collected and centrifuged at 1300 rpm for 5 min and washed with 4 °C ice-cold D-Hanks. Cell suspensions were prepared with binding buffer, and 10mL of Annexin V-APC (eBioscience) was added for staining in the dark. Apoptosis analyses was measured using a FACSCalibur (BD Biosciences). Cell cycle was determined after cells were washed and centrifuged at 1500 rpm for 5 min then fixed in 4 °C precooled 70% ethanol for 1 h. After cells were centrifuged at 1500 rpm for 5 min and washed in buffer, cells were stained by Propidium Iodide (Sigma) solution and analyzed by FACSCalibur (BD Biosciences).

### CCK8 assay

The viability of the lentivirus infected MEC-2 and M01043 cells was assayed using a CCK8 Kit (Sigma). In brief, cells were seeded into five 96-well plates in triplicate (3000–5000 cells/well) and cultured for 24 h, 48 h, 72 and 120 h. Two to four hours before detection, cells were treated with 10 µL of a CCK8 reagent. The OD450 was measured with a Microplate Spectrophotometer (Tecan Infinite).

### Transwell assay

Transwell chambers (Corning) with an 8 μm pore size were precoated with Polycarbonate Matrigel. Lentivirus infected MEC-2 cells were grown in medium without FBS in the upper chamber. Medium supplemented with 30% FBS, 600 µL, was added to the lower chamber. After incubation for 48 h, cells attached to the bottom side of the membrane were fixed by 4% formaldehyde and stained by 400 µL Giemsa. Cells in five random fields were observed, and the migration ability was analyzed.

### Human Apoptosis Antibody Array

The related genes in the human apoptosis signaling pathway were detected after RNA interfered with SYT7 in MEC-2 cells using Human Apoptosis Antibody Array (R&D Systems). After blocking, proteins extracted from lentivirus infected MEC-2 cells were added to the array membranes for incubation overnight at 4 °C and continuing incubation with HRP linked Streptavidin conjugates. Membrane staining intensity was determined using enhanced chemiluminescence (ECL) (Amersham) and the signal densities were analyzed with ImageJ software (National Institute of Health).

### GeneChip analysis

Total RNA was extracted, and the quality and integrity of total RNA was determined by Nanodrop 2000 (Thermo Fisher Scientific) and Agilent 2100 and Agilent RNA 6000 Nano Kit (Agilent). Shanghai Yibeirui Biomedical Science and Technology Co., Ltd. performed the sequencing using Affymetrix Human GeneChip PrimeView according to the manufacturer’s instructions and the outcomes were scanned. Statistical significance assessment of raw data was determined using a Welch t-test with Benjamini-Hochberg FDR (FDR < 0.05 as significant). Enrichment of the canonical signaling pathway and IPA disease and function based on Ingenuity Pathway Analysis (IPA) (Qiagen) was further performed based on the results of RNA sequencing.

### In vivo tumorigenicity experiments and bioluminescence imaging

Twenty female BALB/c nude mice purchased from Beijing Vitalriver Experimental Animal Technology Co., Ltd. were used for in vivo tumorigenicity experiments. The experiment conformed to regulations of the Laboratory Animal Center of Fujian Medical University. Mice were divided into shCtrl and shSYT7 groups and 4 × 10^6^ control and shRNA MEC-2 cells were implanted into 4-week-old mice. Data of animal weight and tumor size were collected 2–3 times a week and tumor volumes were calculated as: V (mm^3^) = L×W^2^ × π/6, where L is the tumor length and W is the tumor width. After 11 days the mice were given 10 µL/g body weight D-Luciferin (15 mg/mL, Shanghai Qianchen) *via* intraperitoneal injection for bioluminescence imaging and the signal was detected using Living Image System (Perkin Elmer). After staining the mice were sacrificed and the tumors were dissected for Ki-67 immunostaining.

### Ki-67 immunostaining

We prepared slides of 2 μm thick tissue section and blocked the sections with 3% H_2_O_2_. All slides were incubated with Ki-67 primary antibody at 4 °C overnight, then incubated with goat anti-rabbit IgG H&L (HRP). Antibodies used in Ki-67 immunostaining are shown in Table S1. All slides were stained by Hematoxylin and Eosin (Baso).

### Statistical analysis

The cell experiments were repeated three times. Data are expressed as the mean ± SD and a Student’s T-Test or one-way ANOVA were used to analyze the statistical significance. A Rank Sum test analysis was used to compare SYT7 expression in lymphoma and normal lymph nodes. The Mann-Whitney U analysis and Spearman Rank correlation analysis were used to analyze the relationship between SYT7 expression and tumor characteristics in patients with CLL. All statistical analysis was performed using SPSS 17.0 (IBM) and GraphPad Prism 6.01 (Graphpad Software) and *P* < 0.05 was considered statistically significant.

## Results

### SYT7 was upregulated in CLL tissues and cells

**Fig. 1:**
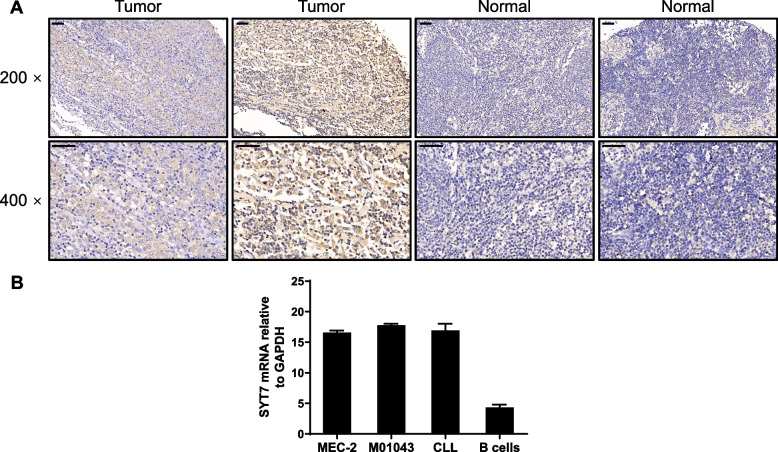
SYT7 is upregulated in CLL tissues and cells. **A** The expression of SYT7 in CLL tissues and corresponding normal tissues was detected by immunohistochemical staining (scale bar = 50 μm). **B** The endogenous expression of SYT7 in CLL cell lines (MEC-2, M01043), primary CLL cells, and normal B cells was detected by qPCR

First, we determined the expression of the SYT7 protein in CLL tissues by IHC analysis. We acquired 179 tumor tissues from patients diagnosed with CLL and 27 normal lymphoid tissues. These tissues were then used to generate a tissue microarray for the detection of SYT7. Using the IHC scores derived from tumor tissues, we divided tissues acquired from patients into an SYT7 high-expression group (98/179 tumor tissues and 7/27 normal tissues) and an SYT7 low-expression group (81/179 tumor tissues and 20/27 normal tissues) (Table 1, *P* < 0.001). Collectively, this data revealed that SYT7 was upregulated in CLL tissues when compared with normal tissues (Fig. 1A). The CLL cell lines exhibited high levels of SYT7 expression (Fig. 1B).

**Table 1:** Expression patterns of SYT7 in lymphoma tissues and normal lymphoid tissues revealed in immunohistochemistry analysis

SYT7 expression	Tumor tissue		Normal tissue	
	Cases	Percentage	Cases	Percentage
Low	81	45.3%	20	74.1%
High	98	54.7%	7	25.9%

*P* < 0.001

### The knockdown of SYT7 inhibited the development of CLL in vitro

**Fig. 2:**
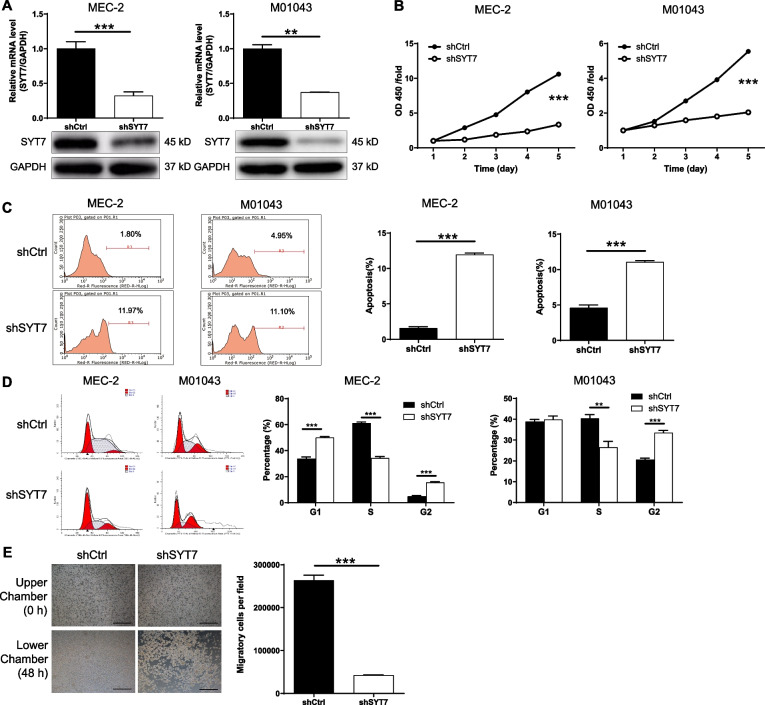
Knockdown of SYT7 inhibits CLL development in vitro. **A** qPCR and western blotting were used to verify the knockdown of SYT7 in MEC-2 and M01043 cells by shSYT7. **B** CCK8 assay was used to verify the inhibited cell proliferation by SYT7 knockdown. **C** Flow cytometry was performed to show the promoted cell apoptosis by SYT7 knockdown. **D** Flow cytometry was performed to show the arrest of cell cycle in G2 phase upon SYT7 knockdown. **E** Transwell assay was utilized to visualize the suppression of cell migration by SYT7 knockdown (scale bar = 400 μm). Data were shown as mean ± SD. **P < 0.01, ***P < 0.001, based on a Student’s T-Test

SYT7 is known to be expressed at high levels in CLL. Consequently, we used GFP-labelled lentiviral vectors to deliver shSYT7 into two CLL cell lines (MEC-2 and M01043) to generate cell lines in which the SYT7 gene had been knocked-down. Fluorescence imaging analysis indicated that the cells had been sufficiently transfected with shSYT7 (Figure S1A). qPCR data are shown in Figure S1B; these data allowed us to identify shSYT7 (RNAi-00148) as the most efficient sequence for subsequent experiments. Both qPCR and Western blotting also demonstrated that the expression of SYT7 were significantly downregulated in shSYT7 cells (Fig. 2A, *P* < 0.001). As predicted, the knockdown of SYT7 led to a significant inhibition in the proliferation of CLL cells, as determined by MTT assays (Fig. 2B, *P* < 0.001). In contrast, flow cytometry showed that the downregulation of SYT7 led to a dramatic increase in the proportion of apoptotic cells (Fig. 2C, *P* < 0.001).

In other experiments, we used a human apoptosis antibody array to identify the differential expression of a range of apoptosis-related proteins upon the suppression of SYT7. These analyses revealed that the expression of p21 was upregulated while the expression of a range of anti-apoptosis proteins were downregulated, including Bcl-2, Bcl-w, cIAP-2, HSP27, IGF-I, IGF-II, IGF-1sR, Livin, sTNF-R1, and TNF-α (Figure S2A, B, *P* < 0.05). In addition, our analyses revealed that SYT7 knockdown not only inhibited the expression of PIK3CA and p-Akt, but also led to a reduction in the expression levels of downstream proteins, including CCND1 and CDK6 (Figure S2C). We also investigated the effects of SYT7 knockdown on the distribution of different phases of the cell cycle; this analysis clearly identified cell cycle arrest in the G2 phase (Fig. 2D, *P* < 0.001).

Transwell assays were also used to investigate the effects of SYT7 suppression on cell migration; these assays revealed that the migration ability of MEC-2 cells was suppressed by the knockdown of SYT7 (Fig. 2E, *P* < 0.001). Collectively, these results showed that the knockdown of SYT7 may result in a significant disturbance in the development of CLL.

### Overexpression of SYT7 promotes CLL development in vitro

**Fig. 3:**
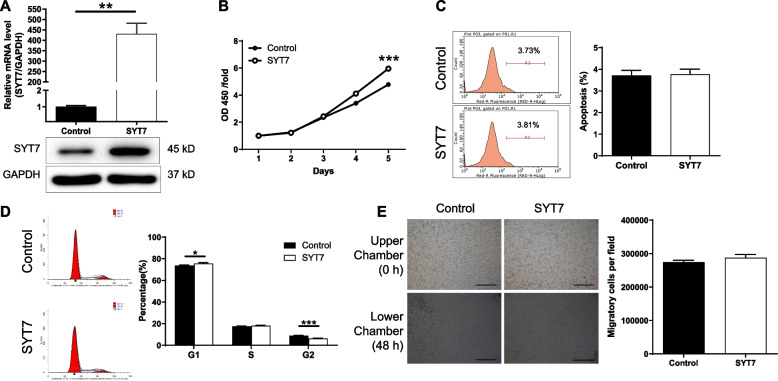
Overexpression of SYT7 promotes CLL development in vitro. **A** qPCR and western blotting were used to verify the overexpression of SYT7 in MEC-2 cells. **B** CCK8 assay was used to verify the promoted cell proliferation by SYT7 overexpression. **C** Flow cytometry was performed to show the effects of SYT7 overexpression on CLL cell apoptosis. **D** Flow cytometry was performed to show the effects of SYT7 overexpression on CLL cell cycle distribution. **E** Transwell assay was utilized to display the effects of SYT7 overexpression on CLL cell migration (scale bar = 400 μm). Data were shown as mean ± SD. *P < 0.05, **P < 0.01, ***P < 0.001, based on a Student’s T-Test

The MEC-2 cells with SYT7 overexpression were also constructed and verified by fluorescence imaging (Figure S3), qPCR and Western blotting (Fig. 3A), further confirming the role of SYT7 in CLL development. As expected, the cells with higher expression of SYT7 showed higher proliferative activity than the ones with relatively lower SYT7 expression (Fig. 3B, *P* < 0.001). However, SYT7 overexpression only showed a slight effect on cell apoptosis (Fig. 3C) which may be attributed to the low apoptotic rate of the cells. As shown in Fig. 3D, ectopic expression of SYT7 decreased the number of cells in G2 phase, which was in contrast with the results of SYT7 knockdown. Unexpectedly, the upregulated SYT7 showed no influence on cell migration (Fig. 3E), the reason of which may be the high motility of MEC-2 cells.

### SYT7 knockdown suppressed the growth of tumors in vivo

**Fig. 4:**
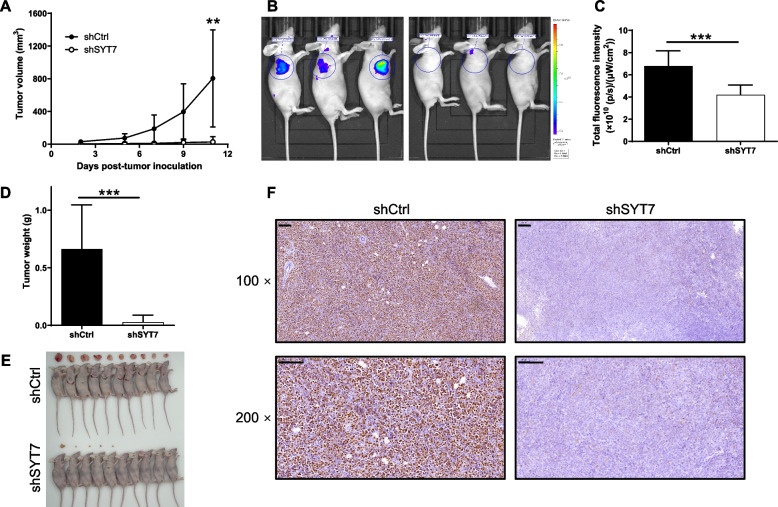
Knockdown of SYT7 suppresses tumor growth in vivo. **A** The tumor growth curve was drawn based on the measurement of tumor volume at indicated time intervals. **B** The in vivo imaging was carried out to evaluate the tumor growth and (**C**) the bioluminescence intensity was scanned and used as a representative of tumor burden in mice xenograft models. **D**, **E** After sacrificing the mice at day 11 post injection, the xenografts were removed and subjected to weighing (**D**) and photographing (**E**). **F** The expression of Ki67 in sections of xenografts was detected by immunohistochemical staining (scale bar = 100 μm). Data were shown as mean ± SD. **P < 0.01, ***P < 0.001, based on a Student’s T-Test

Next, we verified the inhibitory effects of SYT7 knockdown on the development of CLL by constructing a xenograft. This was done by subcutaneously injecting MEC-2 cells with or without the knockdown of SYT7. We then evaluated the xenograft by calculating the tumor volume to create a tumor growth curve, weighing the tumors, and by in vivo imaging of the xenografts. Images acquired from removed xenografts consistently showed that the tumor formed by shSYT7 cells grew much slower than tumors formed by shCtrl cells (Fig. 4A-E). IHC analysis also demonstrated the lower expression levels of Ki67 in tumor sections taken from the shSYT7 group, thus representing lower levels of proliferative activity (Fig. 4F). By carrying out these analyses, we were able to demonstrate the suppressive effect of SYT7 knockdown on the development of CLL.

### SYT7 knockdown inhibited the development of CLL by downregulating KNTC1

**Fig. 5:**
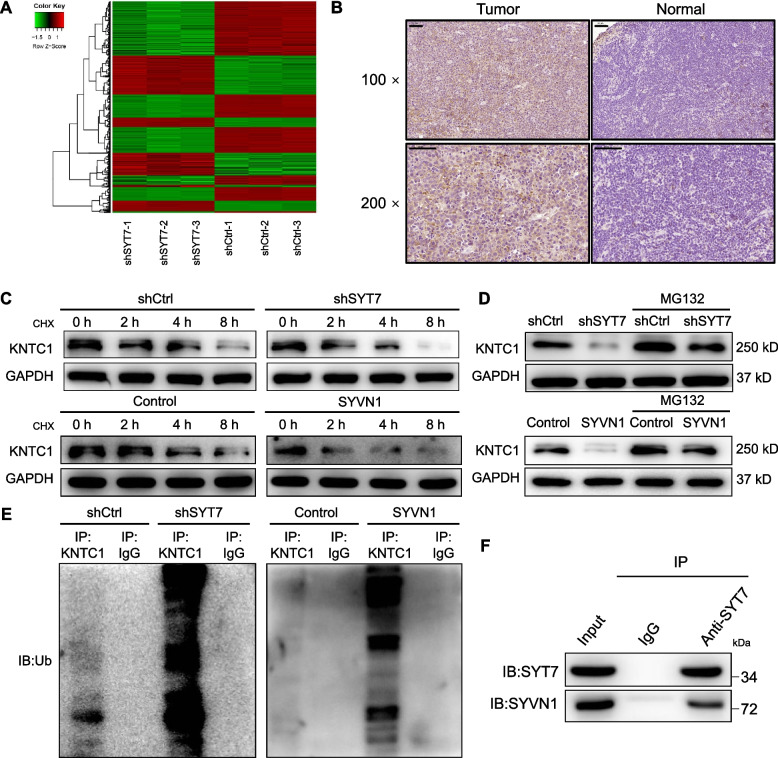
Knockdown of SYT7 inhibits CLL through downregulating KNTC1. **A** The heatmap showed the differentially expressed genes between shSYT7 group and shCtrl group of MEC-2 cells identified by gene microarray. **B** The expression of KNTC1 in CLL tissues and corresponding tissues was detected by immunohistochemistry analysis (scale bar = 50 μm). **C** A chase experiment was performed to evaluate the protein stability of KNTC1 in MEC-2 cells with or without SYT7 knockdown, and cells with or without SYVN1 overexpression. **D** Western blotting was carried out to assess the regulatory effects of SYT7 or SYVN1 on KNTC1 with or without the treatment of proteasome inhibitor MG132. **E** An immunoprecipitation assay was used to visualize the change of ubiquitination of KNTC1 upon SYT7 knockdown or SYVN1 overexpression. **F** A co-immunoprecipitation assay was performed to verify the interaction between SYT7 and SYVN1

Next, we investigated the mechanisms by which SYT7 regulates the development of CLL. To do this, we used a Human Gene Expression Array to create gene expression profiles for normal MEC-2 cells and those in which SYT7 had been knocked down. Using |Fold Change| ≥ 1.5 and FDR < 0.05 as a threshold, we identified 1335 differentially expressed genes (DEGs) in shSYT7 cells, including 532 genes that were upregulated and 803 genes that were downregulated (Fig. 5A and S4A). Enrichment analysis carried out with the IPA database (Figure S4B and C) further identified DEGs showing the greatest changes; these DEGs were then verified by qPCR and Western blotting. There was a significant downregulation of mRNA and protein levels for KNTC1 in shSYT7 cells, thus highlighting the fact that KNTC1 may represent a potential target for SYT7 in the development of CLL (Figure S5A and B). We also used IHC to investigate the expression of KNTC1 in CLL tissues in comparison with normal tissues. We found that levels of KNTC1 were higher in CLL tissues, along with the levels of SYT7 (Fig. 5B). Unexpectedly, a chase experiment indicated that knockdown of SYT7 could apparently decrease the protein stability of KNTC1 (Fig. 5C), which might be the regulatory mechanism of SYT7 on the protein level of KNTC1. In order to explore the possibility that SYT7 affects KNTC1 protein stability through the ubiquitin-proteasome system (UPS), the Ubibrowser tool (http://ubibrowser.ncpsb.org.cn/ubibrowser/) was applied to predict SYVN1 as a promising E3 ligase of KNTC1, overexpression of which could indeed decrease KNTC1 protein stability (Fig. 5C). As expected, the pre-treatment of cells with MG132, a proteasome inhibitor, abolished the effects induced by SYT7 knockdown or SYVN1 overexpression, further indicative of the involvement of UPS (Fig. 5D). Using an immunoprecipitation assay it was demonstrated that both SYT7 knockdown and SYVN1 overexpression enhanced the ubiquitination of KNTC1, which was consistent with the previous results (Fig. 5E). The above results illustrate that SYT7 may upregulate the protein level of KNTC1 through inhibiting SYVN1-mediated KNTC1 ubiquitination. Furthermore, as shown by Fig. 5F, the interaction between SYT7 and SYVN1 may be the pathway by which SYT7 affects SYVN1-mediated KNTC1 ubiquitination.

### Knockdown of KNTC1 inhibits CLL and attenuates the effects of SYT7 overexpression

**Fig. 6:**
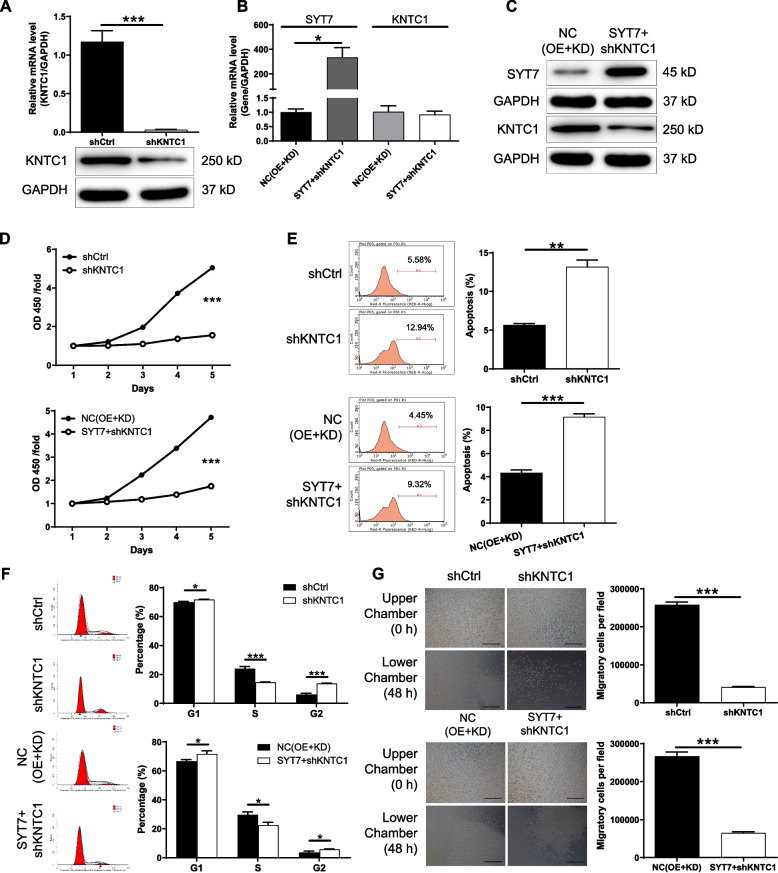
Knockdown of KNTC1 inhibits CLL and attenuates the effects of SYT7 overexpression. **A** The knockdown efficiency of KNTC1 by shKNTC1 in MEC-2 cells was assessed by qPCR. **B**, **C** The mRNA and protein expression of SYT7 and KNTC1 was detected in NC(OE + KD) group and SYT7 + shKNTC1 group of MEC-2 cells by qPCR (**B**) and western blotting (**C**), respectively. **D** CCK8 assay was utilized to show the effects of mere KNTC1 knockdown or simultaneous SYT7 overexpression and KNTC1 knockdown on MEC-2 cell proliferation. **E**, **F** Flow cytometry was utilized to show the effects of mere KNTC1 knockdown or simultaneous SYT7 overexpression and KNTC1 knockdown on MEC-2 cell apoptosis (**E**) and cell cycle distribution (**F**). **G** Transwell assay was performed to show the effects of mere KNTC1 knockdown or simultaneous SYT7 overexpression and KNTC1 knockdown on MEC-2 cell migration (scale bar = 400 μm). Data were shown as mean ± SD. *P < 0.05, **P < 0.01, ***P < 0.001, based on a Student’s T-Test

In order to investigate the synergistic effects of SYT7 and KNTC1 on the development of CLL, MEC-2 cell models with depletion of KNTC1 or simultaneous KNTC1 knockdown and SYT7 overexpression were constructed. As shown in Figure S6, shKNTC1-3 was screened as the most effective construct for silencing KNTC1 and was used in subsequent experiments. Moreover, fluorescence imaging, qPCR, and Western blotting were performed to verify the construction of the cell models. As shown in Figures S7 and 6A-C, KNTC1 was downregulated in both shKNTC1 and SYT7 + shKNTC1 cells, while SYT7 was significantly upregulated in SYT7 + shKNTC1 cells. The subsequent experiments showed that shKNTC1 and SYT7 + shKNTC1 showed similar effects on CLL development, as well as inhibition of cell proliferation (Fig. 6D), promotion of cell apoptosis (Fig. 6E), arrest of cell cycle in G2 phase (Fig. 6F), and suppression of cell migration (Fig. 6G). These results revealed KNTC1 knockdown could attenuate or even reverse the effects of SYT7 overexpression on development of CLL.

## Discussion

The molecular mechanisms driving the pathogenesis and progression of CLL have not been fully elucidated. A subset of patients experience an aggressive disease course in the absence of known high-risk genetic alterations, which indicates that there are still many undiscovered factors promoting the progression of CLL [20]. For example, Hortal et al. recently identified that the overexpression of the wild type oncogene RRAS2 is behind the development of CLL, the most frequent leukemia in the western world [21]. We have also been working on studying the mechanisms of CLL development and applying our results to the treatment of CLL patients [22, 23]. In our study, we found that the overexpression of SYT7 was closely related to the progression of CLL.

We found that SYT7 was upregulated in CLL tissues compared with normal tissues, suggesting that SYT7 may play a critical role in the disease course of CLL. The specific role of SYT7 in CLL was confirmed in further studies by generating SYT7 knockdown and overexpression CLL cell lines. The result of MTT assays revealed that SYT7 knockdown inhibited the proliferation of CLL cells, while flow cytometry result showed that SYT7 downregulation greatly elevated the apoptosis of cells. In particular, the results of an antibody array indicated that anti-apoptosis proteins were decreased after SYT7 knockdown. Other findings also supported the evidence that SYT7 knockdown significantly hampered the development of CLL while the overexpression of SYT7 could promote CLL cell proliferation as expected. The knockdown of SYT7 inhibited xenograft tumor growth of CLL cells in vivo. These lines of evidence all point to SYT7 as promoting CLL progression. Kanda et al. previously reported that SYT7 could drive the development of liver metastasis in gastric cancer and that SYT7 knockdown could attenuate the migration, invasion, and adhesion of gastric cancer cells [17]. Similarly, Liu et al. found that SYT7 could also play a potential tumor promoting role by promoting epithelial-mesenchymal transition in non-small cell lung cancer [18]. Undoubtedly, the mechanisms by which SYT7 functions in CLL are complex, so we further explored the regulatory mechanism of SYT7 to determine how SYT7 can drive CLL progression.

Notably, KNTC1 was confirmed to be the downstream target of SYT7 involved in the progression of CLL after our screening and validation. We confirmed that the expression of KNTC1 in CLL tissue was indeed upregulated, consistent with SYT7. KNTC1, an essential component of the mitotic checkpoint, is mainly involved in spindle assembly and chromosomal segregation [24–26]. KNTC1, which can regulate cell cycle by regulating mitosis, is closely related to the occurrence and development of malignant tumors [27–29]. Studies have shown that KNTC1 can promote the invasion and progression of cervical cancer, hepatocellular carcinoma, and non-small-cell lung cancer [30–32]. Moreover, the high expression of KNTC1 is associated with the poor prognosis of pancreatic cancer patients [33]. In the present study, we found that SYT7 knockdown could apparently decrease the protein stability of KNTC1 and further elucidated that SYT7 could mediate the ubiquitination of KNTC1 through SYVN1. SYVN1 is an E3 ubiquitin ligase involved in endoplasmic reticulum (ER)-associated degradation (ERAD) [34, 35]. The E3 ubiquitin ligase is the main functional enzyme of the ubiquitination system, which mainly transfers ubiquitin from E2 to substrates promoting its degradation [36, 37]. Li et al. have reported that SYVN1 can promote hepatocellular carcinoma tumorigenesis and metastasis by affecting the ubiquitination of proteins such as EEF2K [38]. SYVN1 has also been proved to promote the growth of tumor cells by promoting the ubiquitination and degradation of tumor suppressor SIRT2 [39]. Our study clarified that SYT7 promoted CLL development through inhibiting SYVN1-mediated KNTC1 ubiquitination. Furthermore, KNTC1 knockdown could attenuate or even reverse the effects of SYT7 overexpression on development of CLL, suggesting that SYT7 and KNTCI have a synergistic effect in CLL. Given this synergistic effect, it is worth further exploring whether SYT7 and KNTC1 can be integrated together as molecular markers for CLL disease stratification in the future.

The present study has certain limitations. Due to the biological characteristics of MEC-2 cells with strong self-mobility, the effect of SYT7 overexpression on cell migration was not obvious. In addition, the results of this study still lack clinical data support, and the functional mechanism of SYT7 needs to be verified in primary CLL cells.

In summary, SYT7 regulates the progression of CLL through SYVN1-mediated KNTC1 ubiquitination. The malignant behavior of CLL cells was markedly suppressed after SYT7 knockdown. Additionally, SYT7 and KNTC1 have synergistic effects in promoting the development of CLL. KNTC1 knockdown attenuated the promoting effect of SYT7 overexpression in CLL cells. This study, while contributing to the understanding of the mechanisms of CLL progression, may also provide promising targets for the treatment of CLL.

## Supplementary Information

**Additional file 1: Table S1.** Antibodies used in western blotting and IHC. **Table S2.** IHC scoring criteria. **Table S3. **The target sequences and shRNA sequences. **Table S4.** Primers used in qPCR. **Table S5.** Clinical and pathological characteristics of NHL samples.

**Additional file 2:** **Figure S1. **(A) The transfection efficiencies of shSYT7 and shCtrl in MEC-2 and M01043 cells were evaluated through observing the fluorescence of GFP on lentivirus vector. (B) qPCR was performed to evaluate the knockdown efficiencies of 3 shRNAs targeting SYT7. Data was shown as mean ± SD. *P< 0.05, **P < 0.01.**Figure S2. **(A, B) Human Apoptosis Antibody Array was performed to detect and compare the expression of apoptosis-related proteins in MEC-2 cells with or without SYT7 knockdown. (C) Western blotting was used to detect the protein expression of Akt, p-Akt, CCND1, CDK6 and PIK3CA. **Figure S3. **The transfection efficiencies of Control plasmid and SYT7 overexpression plasmid were evaluated through observing the fluorescence of GFP on lentivirus vector. **Figure S4. **(A) The volcano plot of gene expression profiling in MEC-2 cells with or without SYT7 knockdown. Green dots represent the downregulated DEGs, red dots represent the upregulated DEGs. (B) The enrichment of the DEGs in canonical signaling pathways was analyzed by IPA. (C) The enrichment of the DEGs in IPA disease and function was analyzed by IPA. **Figure S5. **(A, B) A series of differentially expressed genes were selected for further verification by detecting their expression levels by qPCR (A) and western blotting (B), respectively. Data were shown as mean ± SD. **P < 0.01. **Figure S6. **The knockdown efficiencies of 3 shRNAs prepared for silencing KNTC1 were evaluated through qPCR. Data was shown as mean ± SD. *P < 0.05, **P< 0.01. **Figure S7. **The transfection efficiencies of shCtrl, shKNTC1, NC(OE+KD) and SYT7+shKNTC1 plasmids were evaluated through observing the fluorescence of GFP on lentivirus vector.

## Data Availability

The datasets used and/or analyzed during the current study are available from the corresponding author on reasonable request.

## Abbreviations

CLL: Chronic lymphocytic leukemia
SYT7: Synaptotagmin 7
BTK: Bruton’s tyrosine kinase
BCL-2: B-cell lymphoma-2
Co-IP: Co-immunoprecipitation
IHC: Immunohistochemistry
DEGs: Differentially expressed genes
UPS: Ubiquitin-proteasome system
ERAD: Endoplasmic reticulum-associated degradation
