# Supplementary material for: SYT7 regulates the progression of chronic lymphocytic leukemia through interacting and regulating KNTC1
Source: Biomark Res. 2023 Jun 6;11:58. doi: 10.1186/s40364-023-00506-4 (PMC10242988; doi:10.1186/s40364-023-00506-4)
Supplement: Supplementary file 1 — Additional file 1: Table S1. Antibodies used in western blotting and IHC. Table S2. IHC scoring criteria. Table S3. The target sequences and shRNA sequences. Table S4. Primers used in qPCR. Table S5. Clinical and pathological characteristics of NHL samples. [file 40364_2023_506_MOESM1_ESM.pdf]

Table S1 Antibodies used in western blotting and IHC

Primary antibodies	Dilution in WB	Source species	Company	Catalog No.
SYT7	1:1000	Rabbit	Saier Biolabs	SRP09489
GAPDH	1:3000	Rabbit	Bioworld	AP0063
CHSY1	1:1000	Rabbit	biorbyt	orb126811
FUT8	1:500	Rabbit	abcam	ab204124
KNTC1	1:1000	Rabbit	biorbyt	orb101601
PLAC8	1:1000	Rabbit	CST	13885
Akt	1:1000	Rabbit	CST	4685
p-Akt	1:500	Rabbit	R&D	AF887-sp
CCND1 (Cyclin D1)	1:2000	Rabbit	CST	2978
CDK6	1:1000	Rabbit	abcam	ab151247
PIK3CA	1:1000	Rabbit	abcam	ab40776
**Primary antibodies**	**Dilution in IHC**	**Source species**	**Company**	**Catalog No.**
SYT7	1:20	Rabbit	abcam	ab151056
KNTC1	1:100	Rabbit	biorbyt	orb101601
Ki67	1:200	Rabbit	abcam	ab16667

Secondary antibody	Dilution		Company	Catalog No.
HRP Goat Anti-Rabbit IgG (WB)	1:3000		Beyotime	A0208
HRP Goat Anti-Rabbit IgG (IHC)	1:200		Abcam	Ab111909

Table S2 IHC scoring criteria

Staining intensity	Score
Negative	0
Buff	1
Tan	2
Puce	3
**Staining intensity (Proportion of positive cells, %)**	**Score**
0	0
1-24	1
25-49	2
50-74	3
75-100	4
Total score	

Total score: 0-1: negative; 1-4: positive; 5-8: ++ positive; 9-12: +++ positive.

Table S3 The target sequences and shRNA sequences

Gene	No.	Target sequence (5'-3')	shRNA sequences (5'-3')
SYT7	Pbr-00148-a	GCTCACCGTGAAGATCATGAA	CcggGCTCACCGTGAAGATCATGAActcgagTTCATGATCTTCACGGTGAGCTTTTT
SYT7	Pbr-00148-b	GCTCACCGTGAAGATCATGAA	aattcaaaaaGCTCACCGTGAAGATCATGAActcgagTTCATGATCTTCACGGTGAGC
SYT7	Pbr-00149-a	CTGGAACGAGACCTTCCTCTT	CcggCTGGAACGAGACCTTCCTCTTctcgagAAGAGGAAGGTCTCGTTCCAGTTTTT
SYT7	Pbr-00149-b	CTGGAACGAGACCTTCCTCTT	aattcaaaaaCTGGAACGAGACCTTCCTCTTctcgagAAGAGGAAGGTCTCGTTCCAG
SYT7	Pbr-00150-a	GCTCTTGTCTCTCTGCTACAA	CcggGCTCTTGTCTCTCTGCTACAActcgagTTGTAGCAGAGAGACAAGAGCTTTTT
SYT7	Pbr-00150-b	GCTCTTGTCTCTCTGCTACAA	aattcaaaaaGCTCTTGTCTCTCTGCTACAActcgagTTGTAGCAGAGAGACAAGAGC
KNTC1	Pbr-14692-a	TGGGGCATTCGTCTTGGTAAA	CcggTGGGGCATTCGTCTTGGTAAActcgagTTTACCAAGACGAATGCCCCATTTTTg
KNTC1	Pbr-14692-b	TGGGGCATTCGTCTTGGTAAA	aattcaaaaaTGGGGCATTCGTCTTGGTAAActcgagTTTACCAAGACGAATGCCCCA
KNTC1	Pbr-14693-a	TGGTAAATAACTTGCGAGAGT	CcggTGGTAAATAACTTGCGAGAGTctcgagACTCTCGCAAGTTATTTACCATTTTTg
KNTC1	Pbr-14693-b	TGGTAAATAACTTGCGAGAGT	aattcaaaaaTGGTAAATAACTTGCGAGAGTctcgagACTCTCGCAAGTTATTTACCA
KNTC1	Pbr-14694-a	TACTGAATAGATGCAGCTCAA	CcggTACTGAATAGATGCAGCTCAActcgagTTGAGCTGCATCTATTCAGTATTTTTg
KNTC1	Pbr-14694-b	TACTGAATAGATGCAGCTCAA	aattcaaaaaTACTGAATAGATGCAGCTCAActcgagTTGAGCTGCATCTATTCAGTA

Table S4 Primers used in qPCR

Gene	Forward primer sequence (5'-3')	Reverse primer sequence (5'-3')
GAPDH	TGACTTCAACAGCGACACCCA	CACCCTGTTGCTGTAGCCAAA
SYT7	ATCTACCTGCTGCCCGACAA	CCTCTGCACCACCTTCTCA
WNT5A	CGACTATGGCTACCGCTTTG	CTCGTTGTTGTGCAGGTTCAT
PRKACA	GCGCTTTGGGAACCTCAAGA	TGGTAGATGGCAATCCAGTCA
TCF7	GAGACTCTTCCCGGACAAACT	TTGAAGGCGGAGTAGACGGT
MAPK3	ATTGTGCAGGACCTGATGGA	ACGTTGGCGGAGTGGATGTA
FZD1	CTCTTCGTGTACCTGTTTATCGG	TGGTGCCATCGTGCTTCAT
PRKACB	TGGATTGGTGGGCATTAGG	GAACTGAAGTGGGATGGGAAT
WNT3	TTCGGCGTGTTAGTGTCCA	GCCTCGTTGTTGTGCTTGTT
WNT5B	CGCTTCGCCAAGGAGTTTGT	GCCTCGTTGTTTTGCAGGTT
FZD7	ACATCGCCTACAACCAGACC	TTCACCAGCGGGTAGAACTG
PRKAR2A	GCTTTCTCAAGTTCTCGATGCC	CAAAACTGCCACGGTTGTCATA
PLAC8	CACATTTTGTTTCCCGTGCCT	TCATTGCGACGCTTGTTCC
PRKAR2B	GTCAAAGATGGGGAGCATGTAA	GCCGAAACTCCCACGATTA
KIF21B	TGTCGAAGGAGAAGATTGAGGG	GGTGTCCAGGTCGAAGACAAAG
CHSY1	AGTGGGTGGCTTTGATGTTTC	AGGATGGTGGACGTGGACTA
GNG7	ATGTCAGCCACTAACAACATAGCC	ATGAGGTCAGACGCCGCTTT
KNTC1	TGAAACGCTGCTCCACAAC	TGCTCGTCAGTAAAGGAACCAT
MSH6	GTGACTTCTCACCAGGAGATTTG	CACGGACTGATTTCCCTTTCT
KDSR	ACCTGGCTTTGCCGAAGAA	CCATCTGAGCCAAGGGAACT
FUT8	CTTCATCCCCGTCCTCCATA	GACAAACTGAGACACCCACCAC
AKT1	AGGAGGAGGAGGAGATGGACTT	TTGCCCAGCAGCTTCAGGTA

Table S5 Clinical and pathological characteristics of NHL samples.

Variables	Total(N = 189)
Age (years)	
< 70	98 (51.9)
≥ 70	89 (47.1)
Gender	
Male	110 (58.2)
Female	79 (41.8)
ECOG PS score	
0-1	142 (75.1)
2	47 (24.9)
Binet stage (CLL)	
A	48 (25.4)
B	62 (32.8)
C	69 (36.5)
Pathology diagnosis	
CLL/SLL	170 (90.0)
CLL-RT	9 (4.8)
DLBCL	10 (5.2)

Data are shown as n (%). ECOG PS: Eastern Co-operative Oncology Group performance status. CLL: chronic lymphocytic leukemia. RT: Richter transformation. DLBCL: diffuse large B-cell lymphoma.
