# Supplementary material for: SYT7 regulates the progression of chronic lymphocytic leukemia through interacting and regulating KNTC1
Source: Biomark Res. 2023 Jun 6;11:58. doi: 10.1186/s40364-023-00506-4 (PMC10242988; doi:10.1186/s40364-023-00506-4)
Supplement: Supplementary file 2 — Additional file 2: Figure S1. (A) The transfection efficiencies of shSYT7 and shCtrl in MEC-2 and M01043 cells were evaluated through observing the fluorescence of GFP on lentivirus vector. (B) qPCR was performed to evaluate the knockdown efficiencies of 3 shRNAs targeting SYT7. Data was shown as mean ± SD. *P< 0.05, **P < 0.01.Figure S2. (A, B) Human Apoptosis Antibody Array was performed to detect and compare the expression of apoptosis-related proteins in MEC-2 cells with or without SYT7 knockdown. (C) Western blotting was used to detect the protein expression of Akt, p-Akt, CCND1, CDK6 and PIK3CA. Figure S3. The transfection efficiencies of Control plasmid and SYT7 overexpression plasmid were evaluated through observing the fluorescence of GFP on lentivirus vector. Figure S4. (A) The volcano plot of gene expression profiling in MEC-2 cells with or without SYT7 knockdown. Green dots represent the downregulated DEGs, red dots represent the upregulated DEGs. (B) The enrichment of the DEGs in canonical signaling pathways was analyzed by IPA. (C) The enrichment of the DEGs in IPA disease and function was analyzed by IPA. Figure S5. (A, B) A series of differentially expressed genes were selected for further verification by detecting their expression levels by qPCR (A) and western blotting (B), respectively. Data were shown as mean ± SD. **P < 0.01. Figure S6. The knockdown efficiencies of 3 shRNAs prepared for silencing KNTC1 were evaluated through qPCR. Data was shown as mean ± SD. *P < 0.05, **P< 0.01. Figure S7. The transfection efficiencies of shCtrl, shKNTC1, NC(OE+KD) and SYT7+shKNTC1 plasmids were evaluated through observing the fluorescence of GFP on lentivirus vector. [file 40364_2023_506_MOESM2_ESM.pdf]

Supplementary Material

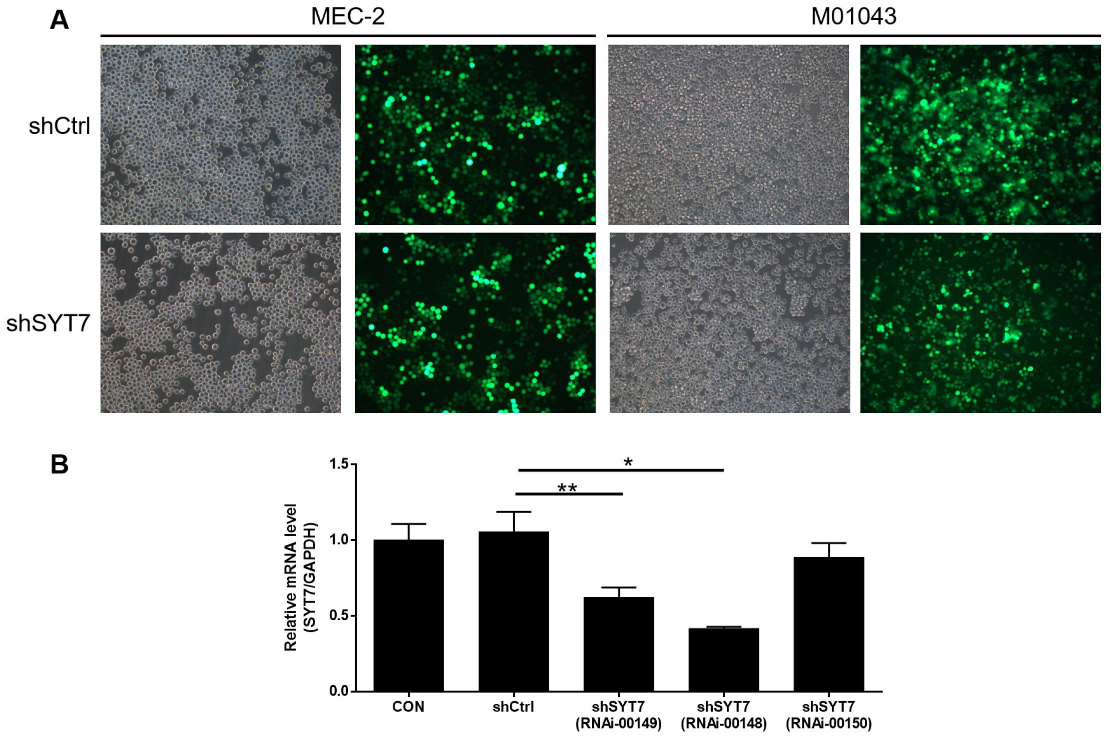

Figure S1. (A) The transfection efficiencies of shSYT7 and shCtrl in MEC-2 and M01043 cells were evaluated through observing the fluorescence of GFP on lentivirus vector. (B) qPCR was performed to evaluate the knockdown efficiencies of 3 shRNAs targeting SYT7. Data was shown as mean ± SD. *$P < 0.05$, **$P < 0.01$

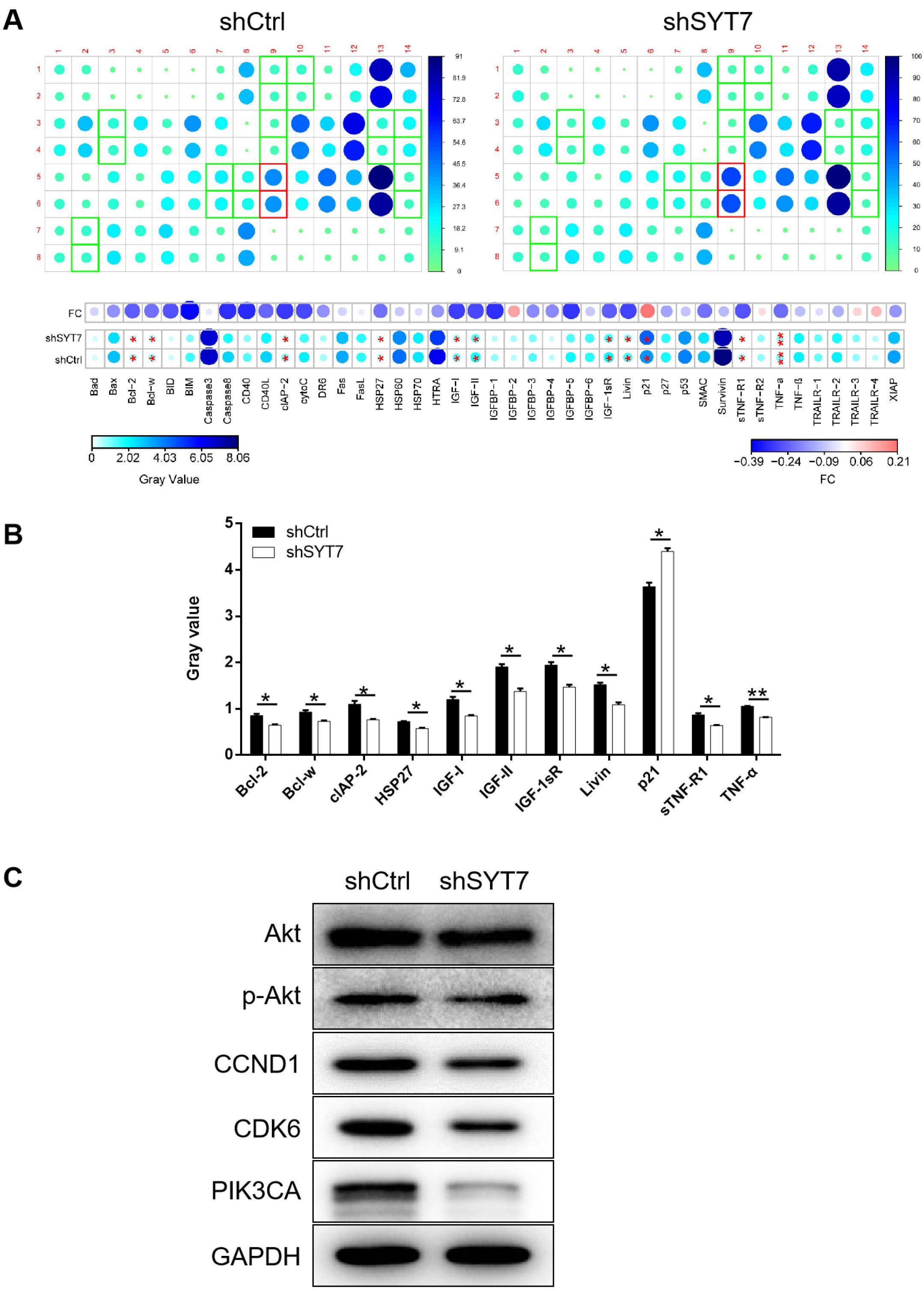

Figure S2. (A, B) Human Apoptosis Antibody Array was performed to detect and compare the expression of apoptosis-related proteins in MEC-2 cells with or without ZNF280A knockdown. (C) Western blotting was used to detect the protein expression of Akt, p-Akt, CCND1, CDK6 and PIK3CA.

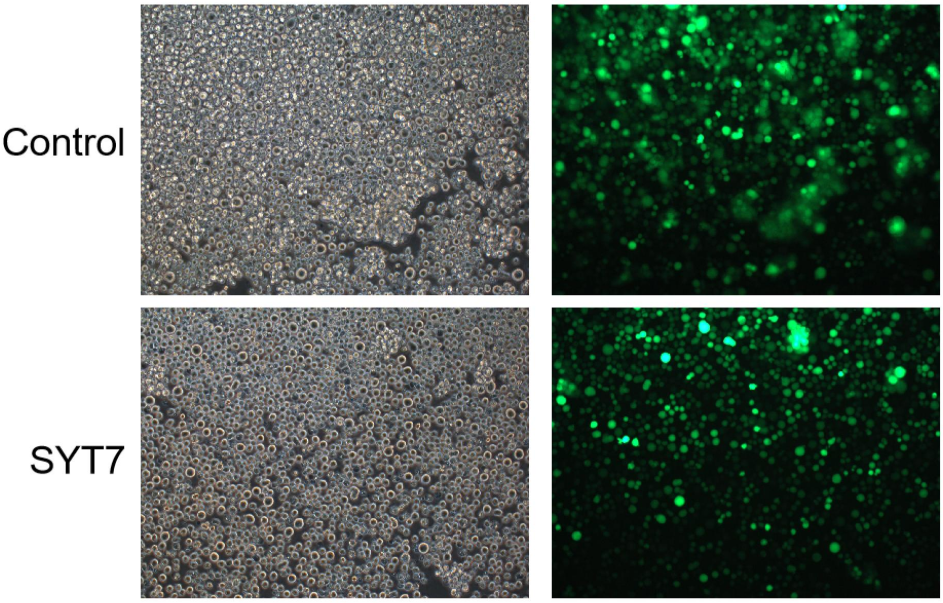

Figure S3. The transfection efficiencies of Control plasmid and SYT7 overexpression plasmid were evaluated through observing the fluorescence of GFP on lentivirus vector.

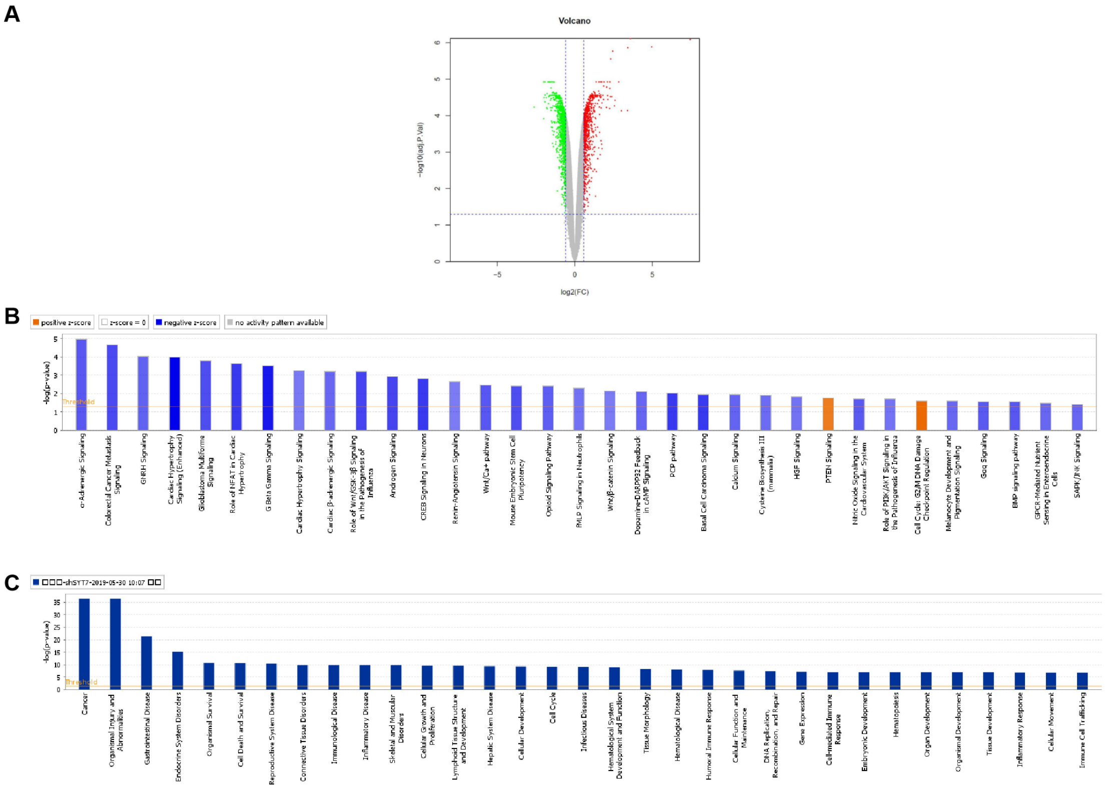

Figure S4. (A) The volcano plot of gene expression profiling in MEC-2 cells with or without SYT7 knockdown. Green dots represent the downregulated DEGs, red dots represent the upregulated DEGs. (B) The enrichment of the DEGs in canonical signaling pathways was analyzed by IPA. (C) The enrichment of the DEGs in IPA disease and function was analyzed by IPA.

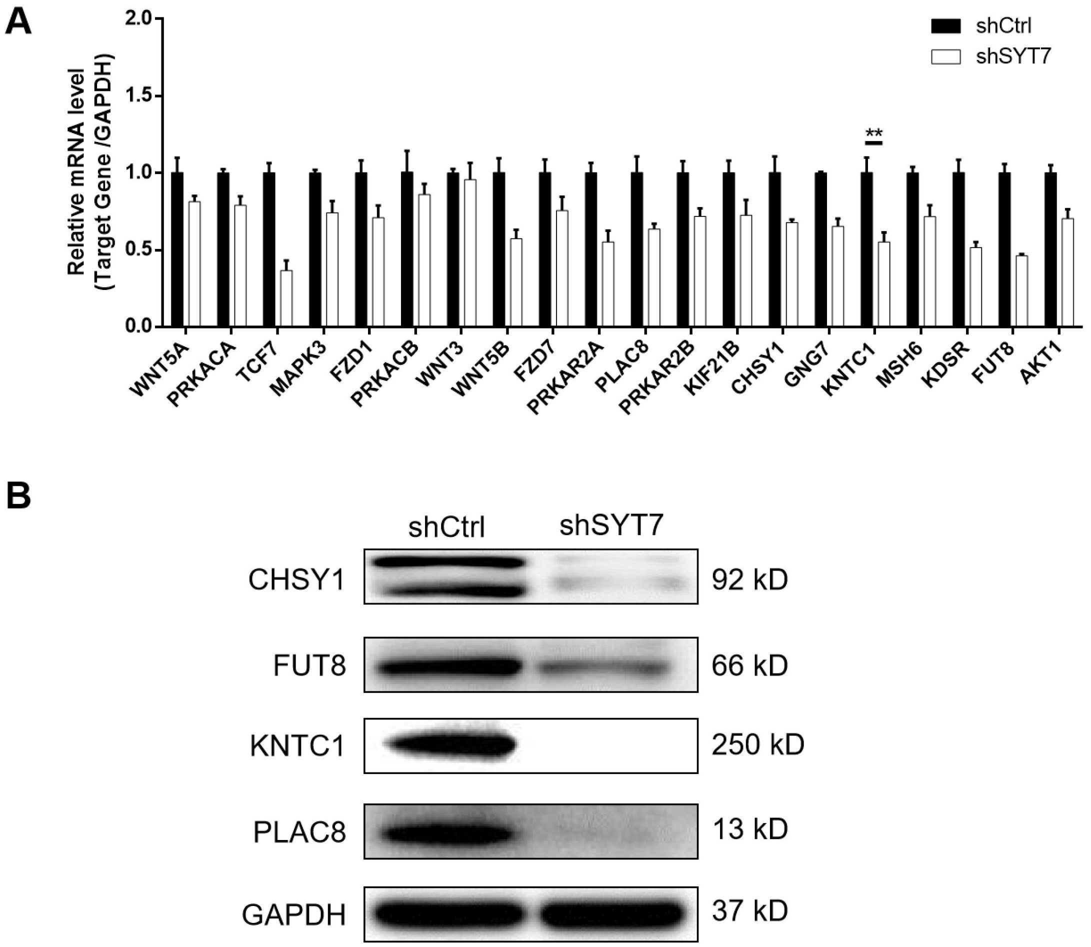

Figure S5. (A, B) A series of differentially expressed genes were selected for further verification by detecting their expression levels by qPCR (A) and western blotting (B), respectively. Data were shown as mean ± SD. $^{**}P < 0.01$

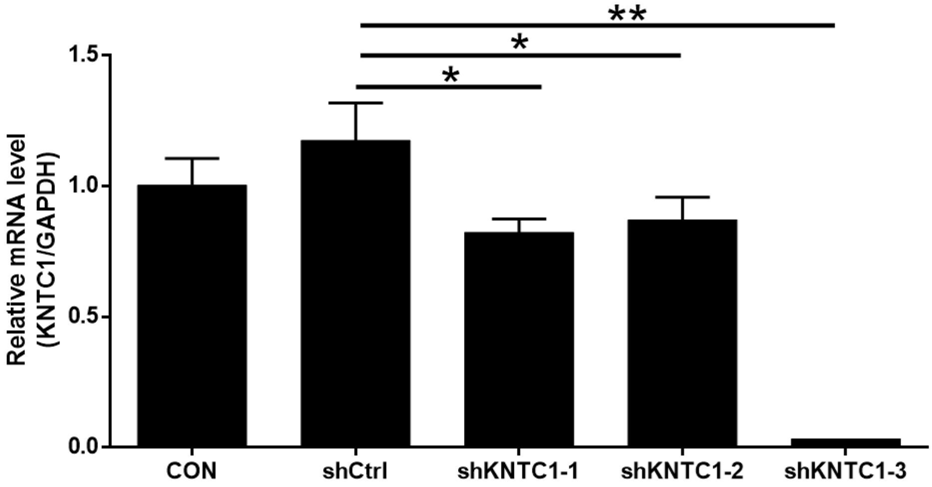

Figure S6. The knockdown efficiencies of 3 shRNAs prepared for silencing KNTC1 were evaluated through qPCR. Data was shown as mean ± SD. **P* < 0.05, ***P* < 0.01

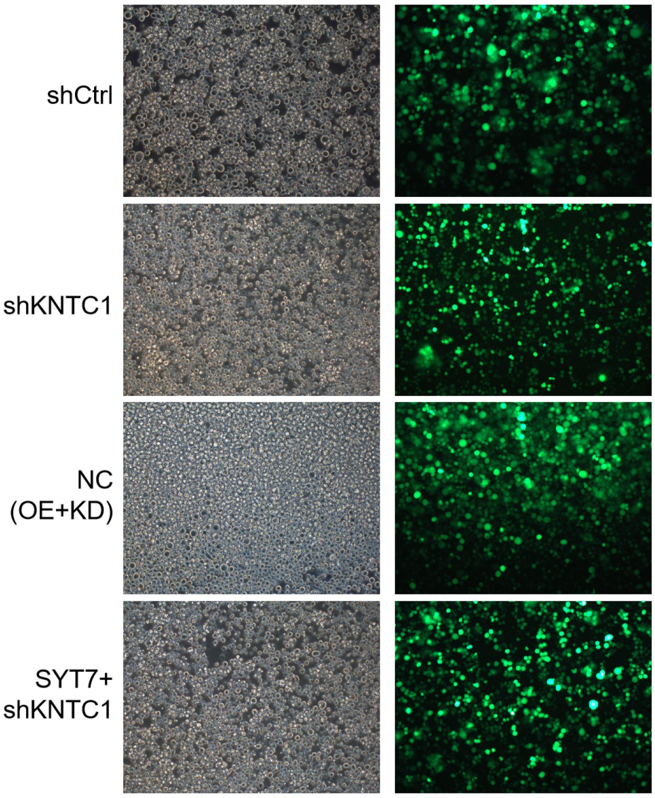

Figure S7. The transfection efficiencies of shCtrl, shKNTC1, NC(OE+KD) and SYT7+shKNTC1 plasmids were evaluated through observing the fluorescence of GFP on lentivirus vector.
